# Tissular Distribution and Metabolism of *trans*-ε-Viniferin after Intraperitoneal Injection in Rat

**DOI:** 10.3390/nu10111660

**Published:** 2018-11-04

**Authors:** Arnaud Courtois, Claude Atgié, Axel Marchal, Ruth Hornedo-Ortega, Caroline Lapèze, Chrystel Faure, Tristan Richard, Stéphanie Krisa

**Affiliations:** 1 Unité de Recherche Œnologie, Molécules d’Intérêt Biologique, EA 4577, USC 1366 INRA, Université de Bordeaux, Bordeaux INP, Institut des Sciences de la Vigne et du Vin, 210 Chemin de Leysottes, 33882 Villenave d’Ornon, France; arnaud.courtois@u-bordeaux.fr (A.C.); axel.marchal@u-bordeaux.fr (A.M.); rhornedo@us.es (R.H.-O.); carolinelapeze@live.fr (C.L.); tristan.richard@u-bordeaux.fr (T.R.); 2 Centre Antipoison et de Toxicovigilance de Nouvelle Aquitaine, Bâtiment UNDR, CHU de Bordeaux, Place Amélie Raba Léon, 33076 Bordeaux, France; 3 Institut de Chimie et de Biologie des Membranes et des Nano-objets (CBMN), Equipe ClipIN (Colloïdes et Lipides pour l’Industrie et la Nutrition) UMR 5248, CNRS, Université de Bordeaux, Bordeaux INP, Bât B14, Allée Geoffroy Saint-Hilaire, 33600, Pessac, France; Claude.Atgie@enscbp.fr (C.A.); chrystel.faure@enscbp.fr (C.F.)

**Keywords:** ε-viniferin, distribution, metabolism, adipose tissue

## Abstract

Background: Recent studies showed that *trans*-ε-viniferin (ε-viniferin), a *trans*-resveratrol dehydrodimer, has anti-inflammatory and anti-obesity effects in rodents. The main purpose of this work was to assess the tissue distribution study of ε-viniferin and its metabolites after intraperitoneal (IP) administration in rat. Methods: After IP injection of 50 mg/kg, ε-viniferin and its metabolites were identified and quantified in plasma, liver, kidneys, adipose tissues, urine, and faeces by Liquid Chromatography-High Resolution Mass Spectrometry (LC-HRMS). Results: ε-Viniferin underwent a rapid hepatic metabolism mostly to glucuronides but also to a lesser extent to sulphate derivatives. The highest glucuronide concentrations were found in liver followed by plasma and kidneys whereas only traces amounts were found in adipose tissues. In contrast the highest ε-viniferin areas under concentration (AUC) and mean residence times (MRT) values were found in white adipose tissues. Finally, much lower levels of ε-viniferin or its metabolites were found in urine than in faeces, suggesting that biliary excretion is the main elimination pathway. Conclusion: A rapid and large metabolism of ε-viniferin and a high bioaccumulation in white adipose tissues were observed. Thus, these tissues could be a reservoir of the native form of ε-viniferin that could allow its slow release and a sustained presence within the organism.

## 1. Introduction

**Figure 1:**
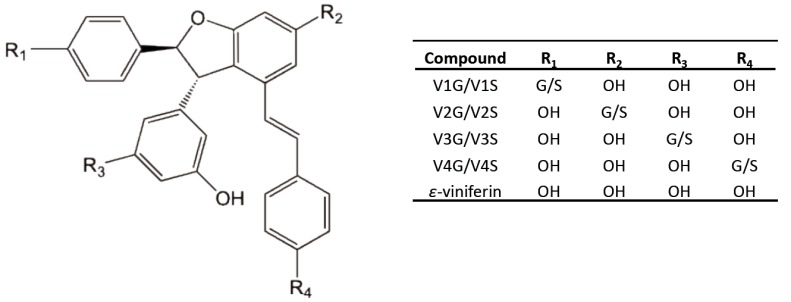
Structural representation of ε-viniferin and its glucuronide and sulphate metabolites. G: glucuronic acid, S: sulphate, OH: hydroxy group.

*Trans*-ε-viniferin (ε-viniferin), a stilbene oligomer formed by two units of *trans*-resveratrol monomer (Figure 1), is found in various plant families such as Vitaceae, Gnetaceae, Cyperaceae, Fabaceae or Dipterocarpaceae [1,2,3,4]. ε-Viniferin is a major constituent of *Vitis* species [5]. In dietary terms, the principal source of ε-viniferin is wine and its amount ranging from 0.2 to 4.3 mg/L [6,7]. It is now considered nowadays as a potential bioactive molecule with promising antioxidant, anti-inflammatory, and cardioprotective activities [8,9,10,11]. 

Recent studies have reported its strong potential anti-obesity effects and that it acts preventively against metabolic syndrome. In fact, ε-viniferin (50 µM) has been demonstrated to be able to inhibit adipogenesis in 3T3-L1 cells by decreasing lipid accumulation and expression of adipogenesis (PPARgamma) and anti-inflammatory (MCP-1) gene markers. In addition, the same authors showed in mice that administration over four weeks of a diet containing 0.2% of ε-viniferin significantly reduced subcutaneous, epididymal, and retroperitoneal adipose tissue weights and consequently the body weight. Moreover, a significantly decrease in inflammatory and obesity-related gene expression (tumor necrosis factor alpha, monocyte chemoattractant protein-1, and leptin) was observed in vitro and in vivo [12]. These results are in accordance with a study in which, ε-viniferin (2.5, 5, and 10 µM) decreased the size of lipid deposits in vitro. It also reduced the body weight and the weight ratio of mesenteric fat in mice (10 and 25 mg/kg/day, 5 weeks) [13]. These findings on the potential anti-obesity properties of ε-viniferin are opening a new and interesting line of research that has received little attention to date.

The persistent shadow of doubt when working with stilbene is its low bioavailability, which is often explained by low absorption and an extensive enteric and hepatic phase II metabolism. Generally, this metabolism results in conversion of the stilbene compound to conjugated metabolites that are rapidly excreted [14,15]. Recently, we described the in vitro metabolism of ε-viniferin using liver fractions. We found an intense metabolism in which more than 75% of the ε-viniferin was converted into glucuronide and sulphate metabolites in rat and human [16]. Mao and co-workers described the pharmacokinetic profile of δ-viniferin, another dehydrodimer of *trans*-resveratrol, in rats [17]. They found a total absorption of 31.5% for δ-viniferin and its glucuronide and sulphate metabolites and reported an absolute oral bioavailability of 2.3% for unchanged δ-viniferin, assuming a high metabolism [17]. Moreover, the apparent volume of distribution value in this study suggested that δ-viniferin and its metabolites could disseminate extensively into organs and tissues.

The biological activity of ε-viniferin may depend highly on its ability to be distributed in target tissues. To our knowledge, the only study that investigated the pharmacokinetic parameters of ε-viniferin was performed in mice [18]. The authors reported a low oral bioavailability of 0.771% and a high IP bioavailability of more than 85%. However, they did not study the metabolism and the tissue distribution of ε-viniferin. 

Given the anti-obesity potential of ε-viniferin and that the onset and evolution of obesity depend on of the regional distribution of adipose tissue, it would be worth exploring the distribution of ε-viniferin and its metabolites in superficial and deep white adipose deposit [19,20]. Thus, the objective of our study was to describe the tissue distribution and excretion of ε-viniferin and its glucuronide and sulphate metabolites after IP injection in rat. A sensitive liquid chromatography-high resolution mass spectrometry (LC-HRMS) Orbitrap mass spectrometer was used to quantify ε-viniferin and its metabolites in plasma, liver, kidneys, adipose tissues, urine and faeces.

## 2. Materials and Methods

### 2.1. Material and Reagents

ε-Viniferin (purity ≥ 95%) was extracted and purified from grapevine shoot extract in our laboratory as described previously [21]. The chemical structure was confirmed by 1H NMR, and high-resolution mass spectrometry [22]. High pressure liquid chromatography (HPLC)-grade acetonitrile, methanol, ethanol, ethyl acetate and formic acid were purchased from Fisher Scientific (Illkirch, France). Ultrapure water used was obtained using a Purelab Ultra System (Elga Lab Water, High Wycombe, UK).

### 2.2. Pharmacokinetic Studies

The study was approved by the Bordeaux University Institutional Ethics Committee for Animal Research (IEC-AR/ MESR approval n°00289.0). All experiments were conducted in accordance with Directive 2010/63/EU on the protection of animals used for scientific purposes. The animals (male Wistar rats) were purchased from Janvier-LABS (Saint-Berthevin, France) (agreement n°A33063917, University of Bordeaux, France). Thirty-six male Wistar rats (250–300 g, 7 weeks old) were maintained in controlled conditions (12 h light/12 h dark cycle, humidity 50–60% and ambient temperature 24 ± 1 °C) and had free access to food and water. ε-Viniferin was dissolved in H_2_O/ethanol (90/10) and administrated intraperitoneally at a dose of 50 mg/kg in a total volume of 1 mL/rat. For the distribution study, rats were sacrificed by a deep anesthesia with isoflurane followed by a rapid exsanguination in random groups of 6 rats at the following periods of time points: 15 and 30 min, and 1, 2, 4 h post-injection. Blood samples (500 µL) were collected in heparinised tubes and plasma was harvested by centrifugation at 4 °C and 10,000 g for 5 min. Rats were then perfused with saline prior to collecting and weighing liver, kidneys, interscapular brown adipose tissue (IBAT) and epididymal (EWAT) and retroperitoneal (RWAT) white adipose tissues. All biological samples were stored at −80 °C until analysis. For the excretion study, 6 rats were housed individually in metabolic cages fitted with urine/faeces separators. Urine and faeces (24 h) were collected after ε-viniferin intraperitoneal IP administration. Urine volumes were measured and faecal samples were weighed. All the samples were rapidly frozen at −80 °C until analysis.

### 2.3. Extraction of ε-Viniferin and its Metabolites from Plasma, Urine and Faeces

An aliquot of 100 µL of plasma was mixed with 300 µL of methanol. After vortex-mixing for 3 min and centrifugation at 12,000 *g* for 30 min at 4 °C, the supernatant was evaporated using a SpeedVac concentrator (Thermo Fisher Scientific, Waltham, MA USA). Four millilitres of urine were vortex-mixed with ethyl acetate (3 mL) for 1 min. After centrifugation at 12,000 *g* for 10 min at 4 °C, the supernatant was recovered and the residue was extracted again using ethyl acetate (3 mL). Both supernatants were combined and evaporated using a SpeedVac concentrator. Faeces were lyophilized, weighed and 100 mg of dried sample were mixed with 800 µL of methanol. The mixture was gently vortexed for 3 min and centrifuged at 12,000 *g* for 10 min at 4 °C. Finally, the supernatant was evaporated. The residue of plasma, urine and faeces extraction was reconstituted in 200 µL H_2_O/methanol (90/10) and filtered through a 0.45 µm PTFE filter (Millex, Darmstadt, Germany) prior to LC-HRMS Orbitrap mass spectrometer analysis.

### 2.4. Extraction of ε-Viniferin and its Metabolites from Tissues

An aliquot of rat tissue (lower than 0.6 g) was homogenized in 3 mL of methanol/H_2_O (80/20) using an Ultra-Turrax homogenizer (IKA, Staufen, Deutschland). Extraction was performed for 1 min in an ultrasonic bath, followed by vortex-mixing for 3 min. After centrifugation at 12,000 *g* for 20 min at 4 °C, the supernatant was recovered and the residue was extracted again using 3 mL of methanol/H_2_O (80/20). Both supernatants were combined, evaporated, reconstituted in 1 mL H_2_O/methanol (90/10) and filtered through a 0.45 µm PTFE filter. Some samples were diluted prior to LC-HRMS Orbitrap mass spectrometer analysis.

### 2.5. LC-HRMS Quantitation of ε-Viniferin and its Metabolites

The LC-HRMS platform consisted of an HTC PAL autosampler (CTC Analytics AG, Zwingen, Switzerland), an Accela U-HPLC system with quaternary pumps and an Exactive benchtop Orbitrap mass spectrometer equipped with a heated electrospray ionization (HESI) probe (both from Thermo Fisher Scientific, Bremen, Germany). For liquid chromatography separation, a C18 column was used as the stationary phase (BEH C18 2.1 mm × 100 mm, 1.7 µm particle size, Waters, Guyancourt, France). The mobile phases were (A) water and (B) acetonitrile both acidified with 0.1% formic acid. The flow rate was 450 µL/min and eluent B varied as follows: 0 min, 25%; 0.5 min, 25%; 3.6 min, 50%; 3.9 min, 50%; 4 min, 100%; 5.7 min, 100%; 5.8 min, 25%; 7 min, 25%. The injection volume was 5 µL. Data were acquired in negative Fourier transform mass spectrometry (FTMS) ionization mode at a unit resolution of 25,000 (m/∆m, full width at half maximum (FWHM) at 200 u). The sheath and auxiliary gas flows (both nitrogen) were optimized at 75 and 18 arbitrary units, respectively. The HESI probe and capillary temperatures were 320 °C and 350 °C, respectively. The electrospray voltage was set to −3 kV, the capillary voltage to −60 V, the tube lens voltage offset to −135 V and the skimmer voltage to −26 V. Mass spectra were recorded from 100 to 1800 Th, with an AGC value of 3.10^6^. Before each series of analysis, the spectrometer was calibrated using Pierce^®^ ESI Negative Ion Calibration solution (Thermo Fisher Scientific).

All data were processed using Qualbrowser and Quanbrowser applications from Xcalibur version 2.1 (Thermo Fisher Scientific, Waltham, MA USA). Peak areas were determined by automatic integration from the extracted ion chromatogram built for the deprotonated ion of ε-viniferin (*m/z* 453.1343 u), ε-viniferin glucuronide (*m/z* 629.1664 u) and ε-viniferin sulphate (*m/z* 533.0912 u) in a 5-ppm window. The ε-viniferin calibration curve was obtained by injections of standard solutions in a range of concentrations varying from 100 ng/L to 2 mg/L showing a good linearity (*R*^2^ = 0.9971) with recovery ratio calculated between 89% and 104%. The quantification limit was set at 10 µg/L according to the methodology described by De Paepe et al. (2013) [23]. Injection of 5 replicates of calibration samples at 10 ug/L and 1 mg/L showed relative standard deviation (RDS) less than 7 and 5%, respectively. Metabolite concentrations were expressed as an equivalent of the ε-viniferin standard curve.

### 2.6. Data Analysis

Variables measured were expressed as mean ± SD of 6 rats for each timepoint. Maximum concentration (Cmax) and time to reach Cmax (Tmax) were recorded directly. Pharmacokinetic parameters such as area under concentration-time curve from time zero to the last point (AUC_0–t_), area under concentration-time curve from time zero to infinity (AUC_0–∞_), elimination half-life (T1/2), and mean residence time (MRT) were analyzed by non-compartmental modelling using the PKSolver program [24].

## 3. Results

**Figure 2:**
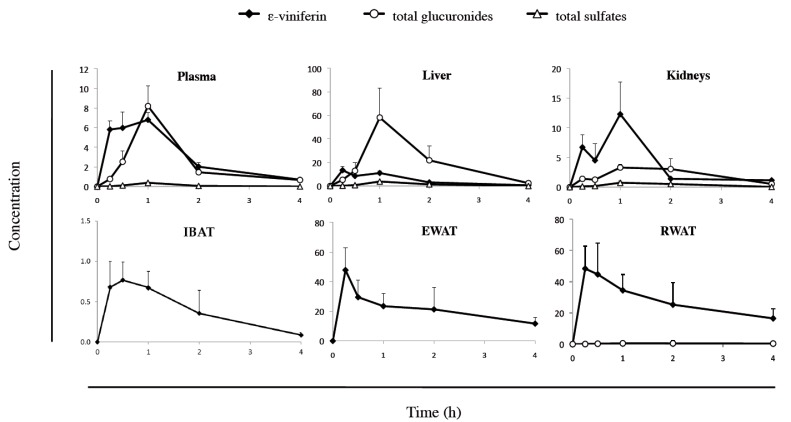
Kinetic profiles of ε-viniferin and its total glucuronide and total sulphate metabolites (*n* = 6). Concentrations were expressed in nmol/mL for plasma and nmol/g for the tissues. Interscapular brown adipose tissue (IBAT), epididymal white adipose tissue (EWAT), retroperitoneal white adipose tissue (RWAT).

The plasma and tissue kinetic profiles of ε-viniferin and its metabolites (Figure 1) were assessed in rats. Animals were administered an IP bolus of 50 mg/kg ε-viniferin and were euthanized at different timepoints (15, 30 min, 1, 2 and 4 h) to obtain blood samples, and to collect the liver, kidneys, IBAT, EWAT and RWAT. ε-Viniferin and its metabolites were identified and quantified by LC-HRMS. The plasma and tissue concentrations of ε-viniferin and its metabolites are illustrated in Figure 2, and the relevant pharmacokinetic parameters are presented in Table 1.

Regarding ε-viniferin, we found a rapid and wide distribution of the native molecule in tissue within the time course examined (Figure 2). Plasma and tissue concentrations of ε-viniferin rapidly increased and reached a maximum concentration between 15 to 60 min after IP injection. The Tmax values for ε-viniferin were 15 min for EWAT and RWAT and liver, followed by IBAT (30 min) and by plasma and kidneys (1 h), as depicted in Table 1. Thereafter, ε-viniferin concentrations rapidly decreased within 4 h in plasma, liver, kidneys and IBAT. Interestingly, 4 h after injection, a high concentration of ε-viniferin still remained in both white adipose tissues (EWAT and RWAT) in which the MRT and T1/2 were the longest (3.86 and 4.13 h; 2.74 and 2.86 h, respectively). These values were more than 2-fold higher for white adipose tissue than for plasma, liver and kidneys (Table 1). Similarly, the highest AUC_0–t_ were found for EWAT and RWAT (84.16 and 109.02 nmol/g×h, respectively), which were from 4- to 8-fold higher than the values for plasma, liver and kidney (12.52, 20.05 and 15.86 nmol/g×h, respectively).

To obtain an accurate description of the pharmacokinetic profile of ε-viniferin in rat, its metabolism was studied. The mass spectra obtained from plasma and tissue analysis showed the presence of ε-viniferin phase-II metabolites: glucuronide and sulphate conjugates. These metabolites, previously characterized in our laboratory, were identified as being V1G, V2G, V3G and V4G for glucuronides and V1S, V2S, V3S, and V4S for sulphates (Figure 1) [16]. The plasma and tissue concentration-time profiles of total glucuronides and of total sulphates are shown in Figure 2.

**Figure 3:**
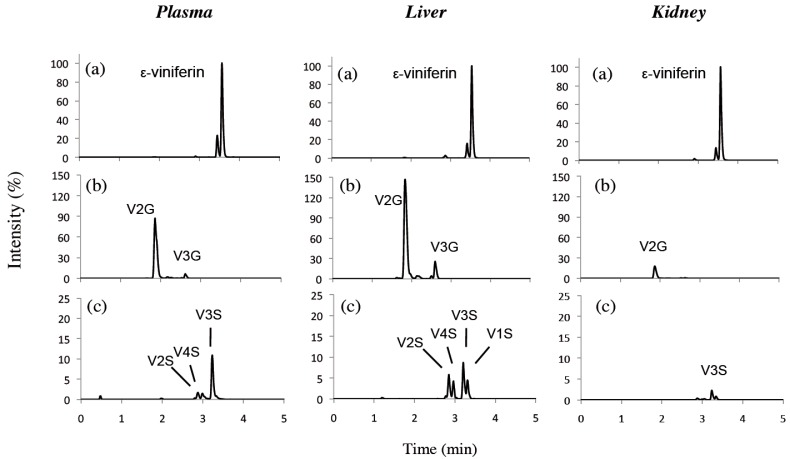
Liquid chromatography-high resolution mass spectrometry (LC-HRMS) extracted ions chromatograms of plasma, liver and kidney extracts from rat 1h after IP administration. (**a**) ε-viniferin (*m/z* 453.1343), (**b**) glucuronide metabolites (*m/z* 629.1664) and (**c**) sulphate metabolites (*m/z* 533.0912). For each tissue, the intensity was expressed relative to the ε-viniferin maximum (%).

Sulphate metabolite levels were lower than those of glucuronide metabolites in plasma and tissues. Glucuronides appeared rapidly in plasma, liver and kidneys and reached a maximum concentration after 60 min (Figure 2). Thereafter, they rapidly decreased both in plasma and liver, but slowly in kidneys. AUC_0–t_ values showed that glucuronides were mainly located in liver, followed by plasma and kidneys (85.01 nmol/mL×h, 10.14 and 8.42 nmol/g×h, respectively) (Table 1). Only traces of glucuronides were detectable in IBAT and EWAT while they were measurable in RWAT. In all tissues and plasma, the major metabolite was V2G, which represented more than 70% of the eight metabolites studied while total sulphates accounted for less than 10%. LC-HRMS chromatograms of ε-viniferin and its metabolites quantified in plasma, liver and kidney are presented in Figure 3.

**Figure 4:**
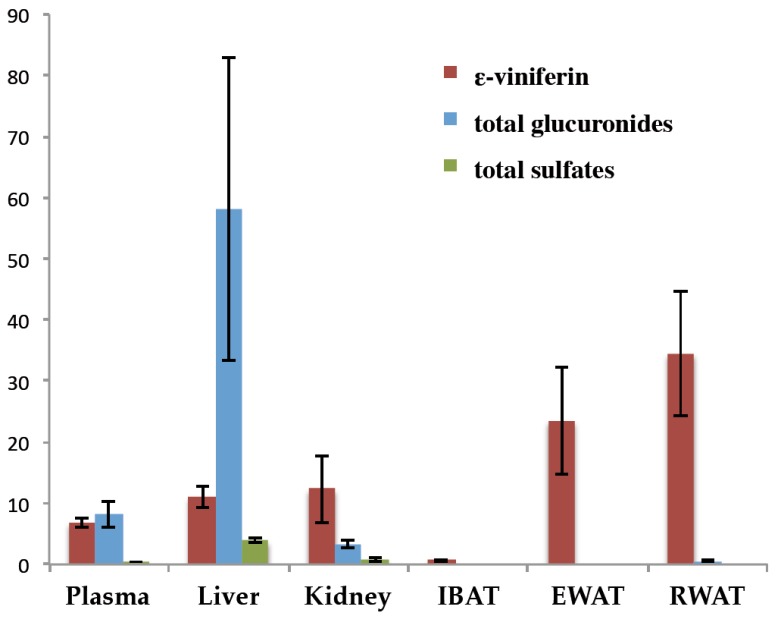
Concentrations ε-viniferin and its total glucuronide and total sulphate metabolites in plasma and organs 1 h after IP administration (*n* = 6). Concentrations were expressed in nmol/mL for plasma and nmol/g for the tissues.

The difference in tissue distribution is highlighted in Figure 4, which shows the 1 h time point (Tmax of ε-viniferin in plasma) for ε-viniferin and its glucurono- and sulpho-conjugates concentrations. Higher ε-viniferin concentrations were found in EWAT and RWAT (23.46 and 34.46 nmol/g, respectively) than in plasma, liver or kidneys (6.80, 11.02 and 12.30 nmol/g, respectively). Regarding glucuronides, the highest concentrations were found in liver followed by plasma and kidneys, whereas only trace amounts were found in adipose tissues. Sulpho-conjugates were found only in very low levels in liver, kidney and plasma.

Urine and faeces were collected 24 h after IP administration of 50 mg/kg ε-viniferin. In urine, ε-viniferin and its metabolites were detected but at less than 0.001% of the administered dose, while 9.7% of the administered dose was excreted in faeces. The major form of dehydrodimer in faeces was unchanged ε-viniferin, with 7.7 ± 1.2% of the initial dose. Glucuronides and sulphates were 0.6 ± 0.2% and 1.4 ± 0.4%, respectively.

## 4. Discussion

After IP injection, the appearance of the administered molecule requires a certain time to reach the systemic blood circulation. In this study, we observed that ε-viniferin was present in the plasma as early as 15 min after administration. Its concentration slowly increased during 1 h and then rapidly declined. This could be due to its rapid biotransformation in the liver (Tmax 15 min). Indeed, ε-viniferin glucuronides appeared as early as 15 min in liver and were present in much greater concentrations than ε-viniferin and the sulphate forms (Table 1). In plasma, kidneys and RWAT, glucuronides were also the main metabolites. Regarding another dehydrodimer of *trans*-resveratrol, δ-viniferin, Mao et al. (2016) showed in vivo that glucuronides were also much more present than sulphates in plasma after oral or intravenous administration. In a previous study using rat liver extracts, we showed that the in vitro metabolism of ε-viniferin produced four glucuronides (V1G, V2G, V3G and V4G) and four sulphates (V1S, V2S, V3S and V4S), with glucuronides being the major metabolites. V2G and V3G represented more than 90% of the total glucuronides, whereas sulphates metabolites were present in equal proportion [16]. We now confirm the same metabolic profile in vivo in rat liver both for glucuronides and sulphates, which is an important finding (Figure 3). Identification of the metabolic profile of ε-viniferin will allow us to perform additional studies into potential values of metabolites.

The AUC0–t for ε-viniferin glucuronides in liver were 8.4- and 10.1-fold higher than those measured in plasma and kidneys, respectively. Moreover, ε-viniferin glucuronides were present in faeces but not in the urine. Therefore, glucuronides were eliminated mainly by the hepato-biliary system, which could explain the low amount measured in plasma, kidneys and urine. Native ε-viniferin was also found in faeces (7.7% of the administered dose), which could be due to its biliary excretion or to the cleavage of glucuronide by glucuronidase in the intestinal lumen. The aglycone and glucuronide forms of *trans*-resveratrol are known to be eliminated mainly in faeces by the bile tract and not in the urine [25,26]. Interestingly, when the bile cannula from a bile-donor rat, which received an oral dose of *trans*-resveratrol, was diverted to the duodenum of a bile-recipient rat, *trans*-resveratrol and its glucuronide were found in the blood-stream of the latter. Therefore, *trans*-resveratrol could undergo an entero-hepatic re-circulation [25]. Moreover, Mao (2016) observed a double peak of δ-viniferin on the concentration-time curve in plasma after oral administration, suggesting the enterohepatic circulation of δ-viniferin [17]. It might thus be of interest to extend the time of exposure of the organism to ε-viniferin and lengthen its pharmacological activity. In urine, the amounts of ε-viniferin, its glucuronides and its sulphates were less than 0.001% of the initial dose, therefore strengthening the notion of an extremely weak urinary elimination. A recent study found that, after an oral dosage, δ-viniferin were eliminated in urine under unchanged, glucuronidated and sulphated forms at a low concentration (<0.05%) [17]. Therefore, urine does not seem to be a major route for eliminating these *trans*-resveratrol dehydrodimers.

In this study we collected two types of white adipose tissues: the epididymal (EWAT) and the retroperitoneal (RWAT), and one brown adipose tissue, the interscapular (IBAT). These two types do not have the same structures or functions. Brown adipose tissue is involved in thermogenesis whereas white adipose tissues regulates energy storage and metabolism through endocrine secretion of adipokines [27]. We report here that, ε-viniferin was found 15 min (Tmax 15 min) after injection in all adipose tissues, indicating a rapid uptake. In EWAT and RWAT, ε-viniferin Cmax were very high, even much higher than the concentration found in the other organs studied. Moreover, ε-viniferin was eliminated slowly, as attested by the high MRT values in EWAT and RWAT (3.83 and 4.13 h, respectively). Based on these results, we speculated that the large amount of the native ε-viniferin found in white adipose tissues could be due to its lipophilic characteristics, as confirmed by its poor aqueous solubility and a high octanol-water ratio partition coefficient (log *p* value 4.60) which promote rapid incorporation in unilocular lipid droplets of adipocytes. Surprisingly, the ε-viniferin concentration in IBAT was very low as compared to that in white adipose tissue. Indeed, brown adipocytes are multilocular with small lipid droplets, surrounded by many mitochondries with a high metabolic activity. This is not conducive to the accumulation of lipophilic compounds that occurs in white adipose tissue.

The very low levels of metabolites detected in each different adipose tissues studied could be due to one of the principal functions of the first pass metabolism, which enhance the hydrophilic characteristics of a compound in order to facilitate its elimination. The low level of glucuronidated and sulphated forms detected in these depots together with the high value of MRT of ε-viniferin suggests that ε-viniferin is not metabolized (or at a very low rate) and that its metabolites are not distributed in white adipose tissues.

Few studies have been conducted on the presence of polyphenols and their metabolites in adipose tissues after administration, and to our knowledge, no one concerning ε-viniferin. It was reported that after oral administration of a grape extract enriched in polyphenols, the unmodified flavanols were the major forms accumulated in mesenteric white adipose tissue. These authors suggested that white adipose tissue acts as a storage compartment for non-metabolized polyphenols [28,29]. To our knowledge, only two studies to date have quantified *trans*-resveratrol in adipose tissues. *Trans*-resveratrol was not present in the adipose tissue of rats, which received it in their diet (30 mg/kg/day) for 6 weeks, while several glucuronide and sulphate metabolites were detected [30]. On the other hand, *trans*-resveratrol and some of its metabolites were detected in pig 6 h after 6.25 mg/kg intragastric administration [31].

In other studies, ε-viniferin decreased the accumulation of lipids and inhibited of the enzyme HMG-CoA reductase, an enzyme involved in cholesterol synthesis, for a concentration range of 2.5 to 50 µM in 3T3-L1 adipocytes [12,13]. Thus, despite the weak adipose concentrations of ε-viniferin observed in our study as compared to those reported to exert an anti-adipogenic effect in vitro, we believe they are sufficient to induce an anti-adipogenic effect in vivo. Indeed, for a given compound, the effective in vitro concentrations are often far higher than the effective in vivo concentrations. Moreover, studies mentioned above also reported that an oral administration of ε-viniferin was found to limit weight gain in mice fed a hypercaloric diet without decreasing their food intake. The authors explained the weight loss by a decrease in the mass of mesenteric white adipose tissue [12,13]. This suggests that the rage of concentrations found in adipose tissue in our study may be responsible for the effect observed in studies in mice. Thus, white adipose tissue may be both a target organ for ε-viniferin and a reservoir of the native form, which might allow its slow release, thereby ensuring that the organism remains exposed to it. This is of interest because this phenomenon could lengthen its biological activity both in adipose tissue and in other organs.

## 5. Conclusions

This is the first demonstration that even if ε-viniferin is highly and rapidly metabolized, it is also bioaccumulated in its native form in white adipose tissues after IP injection in rats. These findings offer a new opportunity to develop innovative strategies to increase the bioavailability of ε-viniferin. Considering its potential in the fight against obesity, this bioaccumulation of ε-viniferin in adipose tissues warrants further investigations. If this lipid-lowering potential were to be confirmed, it would pave the way for new strategies aiming at the prevention or reduction of obesity and related metabolic disorders.

## Figures and Tables

**Table 1:** Pharmacokinetic parameters of ε-viniferin and metabolites in plasma and collected tissues.

Parameters	Plasma	Liver	Kidney	IBAT	EWAT	RWAT
*ε-Viniferin*						
Tmax	1.00	0.25	1.00	0.50	0.25	0.25
Cmax	6.80	13.41	12.30	0.76	47.78	48.32
T1/2	0.95	0.74	1.00	1.01	2.74	2.86
AUC_0-t_	12.52	20.05	15.86	1.58	84.16	109.02
AUC_0-∞_	13.49	20.70	17.53	1.71	130.27	177.02
MRT	1.53	1.24	1.62	1.64	3.86	4.13
*ε-Viniferin Glucuronides*						
Tmax	1.00	1.00	1.00	nq	nq	1.00
Cmax	8.20	58.17	3.30	nq	nq	0.52
T1/2	0.90	0.65	1.08	nq	nq	5.79
AUC_0-t_	10.14	85.01	8.42	nq	nq	1.61
AUC_0--∞_	11.00	87.31	9.27	nq	nq	4.67
MRT	1.66	1.52	2.03	nq	nq	8.81
*ε-Viniferin Sulphates*						
Tmax	1.00	1.00	1.00	nq	nq	nq
Cmax	0.38	3.82	0.74	nq	nq	nq
T1/2	0.49	0.35	0.72	nq	nq	nq
AUC_0-t_	0.44	5.31	1.50	nq	nq	nq
AUC_0-∞_	0.44	5.31	1.55	nq	nq	nq
MRT	1.22	1.37	1.69	nq	nq	nq

Tmax, T1/2 and mean residence time (MRT) were expressed in h. Cmax were expressed in nmol/mL and AUC in nmol/mL×h for plasma, and nmol/g and nmol/g×h for the tissues. nq = non quantified. Interscapular brown adipose tissue (IBAT), epididymal white adipose tissue (EWAT), retroperitoneal white adipose tissue (RWAT).
